# A successful pregnancy following recurrent implantation failure with clinical laboratory strategy

**DOI:** 10.5935/1518-0557.20190093

**Published:** 2020

**Authors:** Roshan Nikbakht, Parvin Dorfeshan, Narjes Dibavand, Afrough

**Affiliations:** 1 Fertility Infertility and Perinatology Research Center, Ahvaz Jundishapur University of Medical Science, Ahvaz, Iran; 2 Department of Social Medicine, Faculty of Medicine, Ahvaz Jundishapur University of Medical Sciences, Ahvaz, Iran; 3 Reproductive Biology research, Infertility Research and Treatment Center of ACECR, Khuzestan, Ahvaz, Iran

**Keywords:** assisted reproductive technology, infertility, *in vitro* fertilization

## Abstract

In treatment cycles of in vitro fertilization (IVF), 15% of the oocytes are immature and in the germinal vesicle (GV) phase. In rare occasions, more than 50% of the oocytes of a patient in a cycle are immature. Selecting fertility treatment for patients in this situation can be very challenging. The patient described in this report was a 35-year-old woman with primary infertility for 10 years. She had undergone fertility treatment six times, with no success. In her cycles, more than 50% of the oocytes were immature and in the GV phase. To address the situation, we developed a coordinated protocol involving medical and embryology procedures, analyzed the patient’s medical records, and looked into the reasons of prior treatment failure. The development of a special and coordinated effort - instead of having medical and embryology teams working in isolation - proved efficient at providing better outcomes to the patient.

## INTRODUCTION

In treatment cycles of in vitro fertilization (IVF), 15% of the oocytes are immature and in the germinal vesicle (GV) phase (Cha & Chian, 1998; Go, 2002). Immature oocytes do not have a fully developed nucleus or cytoplasm. The potential for fertilization and the developmental competence after fertilization of these oocytes is questionable (Chian *et al*., 2004). Immaturity of retrieved oocytes has two potential explanations: one is that heterogeneous follicles at different developmental stages at the start of ovarian hyperstimulation lead to different stages of oocyte maturation (Stouffer & Zelinski-Wooten, 2004); and the other is that in various stages of maturation, aspiration of the small antral follicle during oocyte retrieval may capture oocytes (Triwitayakorn *et al*., 2003). In spite of exogenous gonadotropins in stimulated cycles, it rarely happens that more than 50% of the oocytes are immature.

The patient in this case report was a woman who had had six unsuccessful cycles, in which more than 50% of her oocytes were immature. Selecting fertility treatment for patients in this situation can be very challenging. 

## CASE DESCRIPTION

A couple with primary infertility for 10 years (from 2008 to 2018) came to our fertility clinic in 2018. The patient was a 35-year-old woman with irregular menses (oligomenorrhea), dysmenorrhea, and hirsutism. Her husband was a 42-year-old building contractor who had undergone varicocelectomy. However, sperm analysis showed he had asthenoteratozoospermia (sperm concentration: 20x10^6^, motility: 25% and sperm morphology: 0%).

Within a period of ten years the couple underwent controlled ovulation induction for intracytoplasmic sperm injection(ICSI) six times in two different fertility centers, with no success. In these cycles, a significant proportion of the oocytes were immature and in the GV phase. In all cycles the developed embryos were slow growing, and the patient was unable to get pregnant (Table 1).

The oocytes (GV, MI and MII) retrieved in the cycles were of low quality and all her embryos grew slowly. Frustrations were mounting, as the patient grew tired and anxious. She was thinking about resorting to an egg donor when she came to our center.

The patient arrived at our IVF clinic in July 2018 and said she wanted to try it one last time. During her cycle, a special treatment protocol was developed to treat the patient based on her medical record and past unsuccessful cycles.

### Clinical laboratory strategy

We developed a coordinated medical/embryology protocol for the patient by looking into her medical records and unsuccessful cycles.

The following points were considered in the design of the proposed protocol:

- In ultrasound examination, the patient had at least three follicles measuring more than 18 mm in diameter.
- Being the first patient on that day of ovum pickup (OPU), undergo OPU operation before other patients.
- In the embryology laboratory, the oocytes derived from this patient were dissected as the last patient.
- Being the last patient, her oocytes were injected by sperm in the embryology laboratory.

The patient was placed in the specially designed protocol in November 2018. Her protocol included controlled ovarian stimulation with a gonadotropin releasing hormone (GnRH) antagonist. The cycle was started with Puregon (150 IU/day, MSD, UK). Cetrorelix acetate (Cetrotide, Serono, Geneva, Switzerland) was started on day 5 of stimulation. Pergoveris (150 IU r-hFSH and 75 IU r-Hlh, Merck Serono, UK) was introduced from the day of Cetrorelix acetate injection. When two or more follicles reached a mean diameter of 19-20 mm, ovulation was triggered with hCG (10,000-IU, IBSA) plus one dose of Decapeptyl (0.1mg, Ferring Co., Germany). After 38 hours of hCG injection, transvaginal ultrasound-guided oocyte retrieval was performed. 

In the procedure mentioned above, the oocytes were retrieved at 12:30 p.m. and nine oocytes were received in the corresponding cycle. In this cycle, the numbers of immature and mature oocytes were 4 and 5, respectively (Table 1). The oocytes were denuded and injected with sperm in the embryology laboratory (2:15 p.m.). Two embryos developed after 5 days to the blastocyst stage (the quality of the embryos during growth was graded as A). We made a decision to transfer fresh embryos on day 5 after fertilization. The patient was administered platelet-rich plasma (PRP) on the day before embryo transfer. Her beta hCG test was positive 14 days later. She is currently on the 37^th^ week of gestation.

**Table 1:** Characteristics of all the stimulated cycles that the patient has undergone.

Cycle	Number of Oocytes in the one cycle of treatment	Oocyte nucleus phase (%)			Number of embryo transfer	pregnancy
		GV	MI	MII		
1	12	60	0	40	3	-
2	12	58	9	33	3	-
3	27	74	8	18	3	-
4	12	100	0	0	0	-
5	17	64	7	29	2	-
6	10	100	0	0	0	-
7	9	40	0	60	2	+

## DISCUSSION

Oocyte morphology has been used as a marker to investigate and predict assisted reproductive technology (ART) success. Morphological features of oocytes and embryos are commonly used to determine embryo developmental competence and viability (Gardner *et al*., 2007). Our patient had had low quality oocytes in her previous cycles. Low quality MII oocytes and slow growth embryos relative to the time of fertilization may be related to factors such as oocyte genetic and biochemical disability, which by their turn affect the subsequent development of the embryo (Levran *et al*., 2002; Hartshorne *et al*., 1999). One of the treatment strategies used in these cases is to stimulate the full maturity of immature GV oocytes with in vitro maturation (IVM), a novel technique. However, having normal quality oocytes is a critical factor in the choice of oocytes. Nevertheless, pregnancy rates with IVM are in the range of 30-35% per retrieval with 10-15% implantation rates (Chian *et al*., 2004).

More than 50% of the oocytes harvested from our patient were immature (GV phase), and none of them (GV, MI, and MII) was graded as having good quality during the cycles (grade C). Although egg donors are usually involved in these cases (Bagheri-Lankarani *et al*., 2016; Schattman, 2015; Petropanagos *et al*., 2015), many patients decline this option for psychological, social, or religious reasons.

As a result, since the couple did not accept an alternative treatment, we hoped that by evaluating their unsuccessful cycles we might develop a protocol to improve their chances of success. Furthermore, we hoped that this protocol would decrease the proportion of immature oocytes, enhance fertilization potential, and improve embryo development by providing higher quality mature oocytes.

Follicle and oocyte maturation are time dependent. In order to treat a patient with empty follicle syndrome (EFS), Beck-Fruchter *et al*. (2012) administered a GnRH agonist 40 hours and recombinant hCG 34 hours prior to OPU. In our patient’s case, we prescribed a combination of a GnRH agonist and hCG 38 hours prior to OPU. By using this triggering technique, we accepted the risk of oocyte release with the consent of the patient. The fluid inside the recto-uterine pouch was suctioned and handed over to the embryology laboratory.

Our next strategy was to manage the time for sperm injection. ICSI was performed after OPU. The cells surrounding the oocyte may secrete paracrine substances and growth factors or express adhesion molecules on their surface membranes that might play a critical role in nuclear and oocyte maturation. “For instance, the ovarian brain-derived neurotrophic factor secreted by granulosa and cumulus cells is essential for nuclear and cytoplasmic oocyte development” (Kawamura *et al*., 2005). Other authors postulated that a pre-incubation period between oocyte collection and denudation with two hours between retrieval and denudation and ICSI might not increase the proportion of mature oocytes, but might enhance fertilization and implantation rates (Patrat *et al*., 2012).

We found that dual triggering of final oocyte maturation with a combination of GnRH agonist and hCG, along with a medical/embryology laboratory protocol, might improve the response to stimulated cycles. The increased time between hCG injections and OPU, the increased time between oocyte incubation and granulosa cells (co-culture of oocytes and cumulus cells), followed by the use of ICSI might produce better effects on nuclear and cytoplasmic maturation of the oocyte, with impacts on fertilization potential and development.

## CONCLUSION

This report presented the case of a successful clinical pregnancy of a 35-year-old woman treated based on a coordinated protocol involving medical and embryology procedures. We suggest coordination between clinical and embryology laboratory treatments in these patients, it is needed for achieve the best result in the ART. 
